# circRPS16 Promotes Proliferation and Invasion of Hepatocellular Carcinoma by Sponging miR-876-5p to Upregulate SPINK1

**DOI:** 10.3389/fonc.2021.724415

**Published:** 2021-09-14

**Authors:** Shuwen Lin, Ye Lin, Zhongshi Wu, Wuzheng Xia, Chenglong Miao, Tianyi Peng, Zhen Zhao, Chenggang Ji, Zhikang Mo, Xi Liu, Zhixiang Jian

**Affiliations:** ^1^The Second School of Clinical Medicine, Southern Medical University, Guangzhou, China; ^2^Department of General Surgery, Guangdong Provincial People’s Hospital, Guangdong Academy of Medical Sciences, Guangzhou, China; ^3^Department of General Surgery, Binhaiwan Central Hospital of Dongguan, (Also Called The Fifth People’s Hospital of Dongguan), The Dongguan Affiliated Hospital of Medical College of Jinan University, Dongguan, China; ^4^Department of General Surgery, Pizhou People’s Hospital, Pizhou, China

**Keywords:** hepatocellular carcinoma (HCC), circRPS16, miR-876-5p, SPINK1, proliferation, invasion, ceRNA

## Abstract

The roles of serine protease inhibitor Kazal type 1 (SPINK1) in multiple types of cancers have been significantly documented. However, its specific roles in hepatocellular carcinoma (HCC) remain to be investigated. This study found that SPINK1 is upregulated in HCC and its upregulation correlates with poor prognosis. Besides, functional assays revealed that SPINK1 promotes cell proliferation, cell cycle, and invasion *in vitro*. Through bioinformatics analysis, we speculate that circRPS16 regulates SPINK1 expression by sponging miR-876-5p. This was further verified by the dual-luciferase reporter and fluorescent *in situ* hybridization (FISH) assays. Subsequently, rescue assays verified that circRPS16 promotes cell proliferation, cell cycle, and invasion through miR-876-5p. Importantly, silencing circRPS16 inhibited tumor growth by downregulating SPINK1 expression *in vivo*. Collectively, our results confirm that SPINK1 is a downstream target of circRPS16. Besides, circRPS16 and SPINK1 are oncogenic factors in HCC progression; they provide novel diagnostic and therapeutic targets for HCC patients.

## Introduction

Hepatocellular carcinoma (HCC) is the most prevalent primary liver cancer that has emerged as a critical global medical problem. Furthermore, HCC has ranked the fourth most common cause of cancer-related deaths and the sixth leading type of cancer in terms of incidence (1). Unlike that of the four major cancers, i.e., lung, breast, prostate, and colorectal cancers, whose mortality rates are reportedly declined, the death rate of HCC is still increasing worldwide (2).

Circular RNAs (circRNAs) are a novel class of noncoding RNAs that exert critical regulatory roles in physiological and pathological processes. Unlike the linear RNAs, circRNAs are characterized by covalently closed-loop structures formed through a specific back-splicing mechanism of 5′ and 3′end (3). This specific structure enables circRNAs to tolerate RNase R digestion (4). Therefore, circRNAs have a longer half-life than linear RNA (5). Previous studies have reported abundant and conserved circRNAs across species showing tissue and development-stage-specific expression (6, 7). Based on the above characteristics, circRNAs may serve as potential diagnostic biomarkers and therapeutic molecules. Recent studies revealed the important roles of circRNAs in the progression of HCC. Nonetheless, its molecular mechanisms are not yet fully understood. Therefore, functional exploration of circRNAs may provide a novel diagnostic and therapeutic strategy for HCC.

Serine peptidase inhibitor, Kazal type1 (SPINK1) encodes a 79-amino acid peptide which is located at the chromosomal region 5q32 (8). It belongs to the family of protease inhibitors, also known as tumor-associated trypsin inhibitor (TATI) or pancreatic secretory trypsin inhibitor (PSTI) (9). SPINK1 was originally discovered in the urine of ovarian cancer patients (10). Subsequent studies indicate that SPINK1 has been detected in multiple types of cancers including bladder, renal, colorectal, prostate, and liver cancers (8, 11). For HCC, high SPINK1 expression acts as prognostic and diagnostic biomarkers that promote cell proliferation and metastasis (12, 13). This denotes the important roles of SPINK1 in HCC progression. Nonetheless, the regulatory mechanisms promoting the aberrant expression of SPINK1 remain to be investigated.

Herein, we found SPINK1 upregulation in HCC tissues and its high expression was closely associated with poor prognosis of HCC patients. Notably, functional assays revealed the oncogenic roles of SPINK1. Through bioinformatics analysis, we predicted that circRPS16 may regulate SPINK1 expression by sponging miR-876-5p; this was confirmed by mechanistic investigations and rescue assays. Additionally, *in vivo* assays demonstrated that circRPS16 knockdown inhibited tumor growth by inhibiting SPINK1 expression. In summary, our research provides potential diagnostic and therapeutic biomarkers for HCC patients.

## Materials and Methods

### Bioinformatic Analysis

The microarray dataset (GSE14520) (14) analyzing gene expression patterns in tumor and paired nontumor tissue of HCC patients was downloaded from the Gene Expression Omnibus (GEO) database (https://www.ncbi.nlm.nih.gov/gds). The heatmap of the differentially expressed genes was performed using R studio. Kaplan-Meier Plotter (http://kmplot.com/analysis) was used to investigate the significance of SPINK1 expression and the survival of HCC patients (15). The miRWalk 2.0 (http://zmf.umm.uni-heidelberg.de) and starBase (http://starbase.sysu.edu.cn/) databases were used to predict the competing endogenous RNAs (ceRNA) networks (16). The immunohistochemistry (IHC) results of SPINK1 in normal and HCC tissues were obtained from the Human Protein Atlas (http://www.proteinatlas.org) (17).

### Tissue Specimens and Cell Lines

A total of 25 paired HCC and adjacent noncancer tissues obtained from patients of the Guangdong Provincial People’s Hospital, Guangdong Academy of Medical Sciences (Guangdong, China) were used to perform quantitative reverse transcription PCR (qRT-PCR) assays. All the specimens were confirmed through pathological examination. The patients signed the informed consent before surgery. This study was reviewed and approved by the Research Ethics Committee, Guangdong Provincial People’s Hospital, Guangdong Academy of Medical Sciences. The specimens were preserved in liquid nitrogen after resection. Two HCC cell lines (Huh7 and HepG2) were procured from the American Type Culture Collection (ATCC). The cells were regularly cultured in Dulbecco’s modified Eagle’s medium (DMEM) with 10% fetal bovine serum (FBS) and 1% penicillin-streptomycin (Gibco Invitrogen, Grand Island, NY, USA) in a humidified 5% CO_2_ atmosphere at 37°C.

### RNA Extraction and qRT-PCR

Total RNA was extracted from HCC tissues using TRIzol reagent (Invitrogen, Camarillo, CA, USA) following the manufacturer’s instruction; exactly 1 ml TRIzol was used per 30 mg of HCC tissue or 10^6^ cells. After the concentration determination, the total RNA was stored at −80°C to prevent degradation. For qRT-PCR, PrimeScript RT reagent kit (Takara, Kusatsu, Japan) and an SYBR Premix Ex Taq II (Takara) were used for SPINK1 and circRPS16. β-Actin was used as an endogenous control. Meanwhile, Mir-X™ miRNA First-Strand Synthesis Kit (Takara) and SYBR^®^ Premix Ex Taq™ II (Takara) were used for miR-876-5p, and RNU6-2 was used as an endogenous control. RiboBio (Guangzhou, China) synthesized the bulge-loop miRNA qRT-PCR primer sets specific for miR-876-5p. Sangon Biotech (Shanghai, China) synthesized the SPINK1 and circRPS16 primers. All the procedures were conducted according to the manufacturer’s instructions. Primers for circRPS16: 5′ GCCCATCGTGACTCAAAACT 3′ (forward) and 5′ TTTTGGACTCGCAGCG AC 3′ (reverse). Primers for miR-876-5p included 5′ CGCGTGGATTTCTTT GTGAATCACCA-3′. Primers for SPINK1 included 5′ CCTGTCTGTGGGACTGATGGAAATAC 3′ (forward) and 5′ TGAATGAGGATAGAAGTCTGGCGTTTC 3′ (reverse). Primers for RNU6-2: 5′ TGGCACCCAGCACAATGAA 3′ and 5′ CTAAGTCATAG TCCGCCTAGAAGCA 3′ (reverse). Primers for β-actin: 5′ CCTGGCACCCAGCACAAT 3′ (forward) and 5′ GGGCCGGACTCGTCATAC 3′ (reverse).

### Transfection

Lentiviral-circRPS16-RNAi (Hanbio, Shanghai, China) was transfected to inhibit cicRNA RPS16 expression (10 μl, 1 × 10^8^ TU/ml). circRPS16 siRNA (RiboBio, Guangzhou, China), (5 μl, 20 μM), circRPS16 overexpression plasmid (Hanbio, Shanghai, China) (2 μg), miR-876-5p mimics (RiboBio, Guangzhou, China) (5 μl, 20 μM), miR-876-5p inhibitor (RiboBio, Guangzhou, China) (5 μl, 20 μM), SPINK1 siRNA (RiboBio, Guangzhou, China) (5 μl, 20 μM), and SPINK1 overexpression plasmid (Hanbio, Shanghai, China) were obtained for transient transfection. The Lipofectamine 3000 (Invitrogen, Camarillo, CA, USA) was used for transfection following the manufacturer’s instructions. The siRNA sequences against SPINK1 included 5′ TGGCCCTGTTGAGTCTATCTGGTAA 3′ (sense), sequence 5′ TTACCAGATAGACTCAACAGGGCCA′ (antisense). Meanwhile, siRNA sequences against circRPS16 included 5′ TTCAAGAAATGTGGATGAG 3′ (sense), 5′ CTCATCCACATTTCTTG AA 3′ (antisense).

### Protein Extraction and Western Blot

Protein lysates were extracted using RIPA (Beyotime Institute of Biotechnology, Shanghai, China) following the manufacturer’s instructions. The concentrations of protein lysates were qualified by the BCA kit (Beyotime Institute of Biotechnology, Shanghai, China). Protein lysates were separated in 10% SDS-PAGE gel then transferred to polyvinylidene fluoride membranes (Millipore, Billerica, MA, USA). After incubation of primary and secondary antibodies, the belts were visualized under enhanced chemiluminescence reagents. The primary antibodies included anti-SPINK1 at dilution rate of 1:5,000 (Abcam, Shanghai, China), anti-β-actin at dilution rate of 1:5,000 (Proteintech, Wuhan, China), and secondary antibody at dilution rate of 1:2,000 (Proteintech, Wuhan, China).

### CCK-8 Assay

Cell Counting Kit-8 (CCK-8) was purchased from Dojindo (Shanghai, China). Based on the manufacturer’s instructions, 5,000 cells were seeded into 96-well plates, 4 h before detection, then 10 μl CCK-8 solution was added to each well. The OD values at 24, 48, 72, and 96 h were recorded for subsequent analyses.

### EdU Assay

The EdU kit was purchased from Ribobio (Guangzhou, China), and the assay was conducted based on the manufacturer’s instructions. In the present study, 10^5^ cells were seeded into 96-well plates and incubated with EdU regents for 2 h. After fixation with 4% paraformaldehyde, the cells were incubated with Appllo^®^ regents. The Hoechest^®^ was then used for DNA dyeing. The pictures were collected with a fluorescence microscope.

### Flow Cytometry

Flow cytometry was used to analyze the cell cycle. After transfection, HCC cells were fixed with 70% ethanol and stained with propidium iodide (PI) (Kaiji, Nanjing, China). Thereafter, the fixed cells were analyzed using a flow cytometer (Beckman FC500, Los Angeles, CA, USA).

### Transwell Assay

The invasion assay was performed using Matrigel Invasion Chambers (8 μm) (MilliporeSigma, Burlington, MA, USA) following the manufacturer’s instructions. Exactly 20,000 cells were seeded onto the upper chambers with serum-free DMEM; then DMEM with 10% FBS was added to the lower chambers. After 24 h incubation, cells on the upper surface of the insert chamber were eliminated. Cells migrating to the bottom of the insert membrane were fixed with 4% paraformaldehyde and stained with crystal violet.

### Fluorescence *In Situ* Hybridization

Cell climbing was fixed in 4% paraformaldehyde. Then, proteinase K was used for digestion. After blocking with rabbit serum, hybridization in HCC cells were performed overnight using miR-876-5p, circRPS16 probes. Specimens were analyzed using positive fluorescence microscope. The miR-876-5p probe for fluorescent *in situ* hybridization (FISH) was 5′-TGGTGATTCACAAAGAAATCCA-3′, whereas the circRPS16 probe for FISH was: 5′-AGCCTCATCCACATTTCTTGAAACTTTAA-3′.

### Dual-Luciferase Reporter Assay

For the validation of circRPS16 and miR-876-5p combination, the wild and mutant types of the circRPS16 were directly synthesized into the psi-check2 vector. Then, 0.16 µg circRPS16 wild/mutant-type vector and 5 pmol miR-876-5p mimics/NC were cotransfected. For the validation of miR-876-5p and SPINK1 combination, the wild and mutant types of the 3′UTR sequence of SPNK1 were directly synthesized into the psi-check2 vector. Subsequently, 0.16 μg plasmid comprising the wild/mutant types of SPINK1-3′UTR and 5 pmol miR-876-5p mimics/NC were cotransfected. After 48 h, firefly luciferase (internal reference) and Renilla luciferase activities were measured using the Promega Dual-Luciferase system.

### Animal Experiment

Four-week female nude mice were obtained from the Peking University Animal Center (Beijing, China). After acclimatization for 1 week, 2 × 10^6^ HepG2 cells transfected with either Lentiviral-circRPS16-RNAi or negative control (NC) were subcutaneously injected into the right back of each mouse. After four weeks, the mice were killed and tumor weights were measured then recorded in grams. The animal study was reviewed and approved by the Institutional Animal Care and Use Committee of the Guangdong Provincial People’s Hospital, Guangdong Academy of Medical Sciences.

### Immunohistochemistry

The tumor tissues were fixed by 4% paraformaldehyde. After paraffin embedding and pathological section, the slides were incubated with primary antibodies overnight at 4°C and then incubated with secondary antibodies at room temperature for 2 h. The expression was evaluated using a composite score obtained by multiplying the values of staining intensities (0, no staining; 1, weak staining; 2, moderate staining; 3, strong staining) and the percentage of positive cells (0, 0%; 1, <10%; 2, 10%–50%; 3, >50%). IHC kit was purchased from MXB^®^ (Fuzhou, China), and the experiment was performed under the manufacturer’s instructions.

### Statistical Analysis

Statistical analysis was performed using GraphPad Prism 7 software. Quantitative data were presented as mean ± standard deviation (SD). The paired or unpaired *t*-test was used for quantitative data. Differences with *p*-values (*p < 0.05; **p < 0.01; ***p < 0.001) were considered statistically significant.

## Results

### Upregulation of SPINK1 in HCC Is Associated With Poor Prognosis

**Figure 1:**
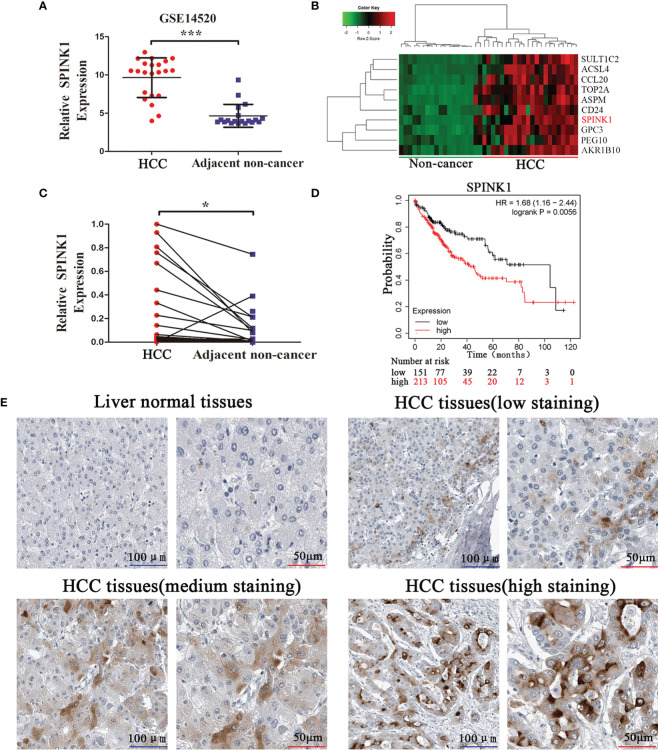
SPINK1 is upregulated in HCC tissues, and high SPINK1 expression is associated with poor prognosis. **(A)** GEO dataset analysis (GSE14520) reveals that SPINK1 was upregulated in HCC tissues. Data are presented as mean ± SD; statistical significance was assessed by Student’s t-test. ***p < 0.001. **(B)** The heatmap showed that SPINK1 was primarily clustered in HCC tissues. **(C)** The results of qRT-PCR reveal that SPINK1 was upregulated in HCC tissues. Data are presented as mean ± SD; statistical significance was assessed by paired *t*-test. *p < 0.05. **(D)** Kaplan-Meier analysis of overall survival from the TCGA dataset reveals that high SPINK1 expression was associated with poor prognosis; the log-rank test was used. *p* = 0.0056. **(E)** Immunochemistry (IHC) results from Human Protein Atlas reveal that SPINK1 was upregulated in HCC tissues.

Based on the GSE14520 dataset, we found that the SPINK1 mRNA levels were significantly upregulated in tumor samples (**Figure 1A**). The heatmap illustrated that SPINK1 was highly enriched in tumors (**Figure 1B**). Furthermore, qRT-PCR including 25 paired HCC and adjacent nontumor tissues confirmed that SPINK1 was upregulated in HCC tumors (**Figure 1C**). Analysis of The Cancer Genome Atlas (TCGA) using Kaplan-Meier plotter (18) revealed that high SPINK1 expression was closely related to low survival rate (**Figure 1D**). The IHC results from the Human Protein Atlas showed that SPINK1 protein was upregulated in HCC tissues and almost undetected in liver normal tissues (**Figure 1E**). Collectively, these findings suggest that SPINK1 is upregulated in HCC tissues and closely associated with the poor prognosis of HCC patients.

### SPINK1 Exerts Oncogenic Roles of HCC *In Vitro*

**Figure 2:**
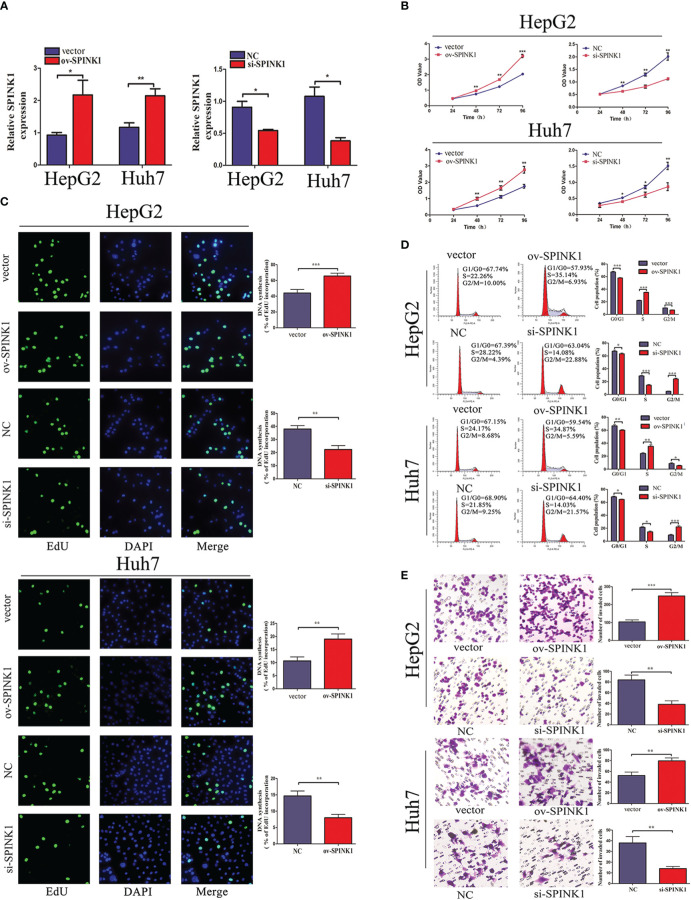
SPINK1 regulates HCC cell proliferation, cell cycle, and invasion capacity. **(A)** The qRT-PCR results reveal the SPINK1 expression level after transfection of SPINK1 overexpression vector or siRNA. Independent experiments were repeated three times; data are presented as mean ± SD; statistical significance was assessed by paired *t*-test. *p < 0.05, **p < 0.01. **(B)** CCK-8 and **(C)** EdU assays reveal SPINK1 overexpression (ov) promotes and knockdown (si) inhibits the proliferation of HCC cells. Independent experiments were repeated three times; data are presented as mean ± SD; statistical significance was assessed by Student’s *t*-test. *p < 0.05, **p < 0.01. **(D)** Flow cytometry shows SPINK1 overexpression accelerates cell cycle, while, SPINK1 knockdown induces cell cycle arrest. Independent experiments were repeated three times; data are presented as mean ± SD; statistical significance was assessed by Student’s *t*-test. *p < 0.05, **p < 0.01, ***p < 0.001. **(E)** Transwell assays revealed SPINK1 overexpression promoted and its knockdown attenuated the invasion of HCC cells. Independent experiments were repeated three times; data are presented as mean ± SD; statistical significance was assessed by Student’s *t*-test. **p < 0.01, ***p < 0.001. ov, overexpressed plasmid vector; si, siRNA; NC, negative control.

Furthermore, the functions of SPINK1 were investigated *in vitro*. To downregulate or upregulate the SPINK1expression, siRNA or overexpressed plasmid vector and the corresponding NC were respectively transfected into HCC cell lines, HepG2 and Huh7. First, the transfection efficiency was examined through qRT-PCR. siRNA transfection downregulated SPINK1 expression, whereas overexpressed plasmid transfection upregulated it (**Figure 2A**). CCK-8 and EdU incorporation assays revealed that SPINK1 knockdown inhibited the cell proliferation and overexpression of SPINK1 promoted cell proliferation (**Figures 2B, C** **)**. Furthermore, flow cytometry analysis showed that downregulation induced cell cycle arrest, while overexpression of SPINK1 accelerated the cell cycle (**Figure 2D**). Transwell assay was performed to find out whether SPINK1 regulates the invasion ability of HCC cells. Consequently, SPINK1 overexpression stimulated cell invasion, while its downregulation demonstrated the opposite effect (**Figure 2E**). Together, these results indicate that SPINK1 exerts important oncogenic roles in cell proliferation, cell cycle, and invasion of HCC cells *in vitro*.

### circRPS16 Regulates SPINK1 *via* miR-876-5p

**Figure 3:**
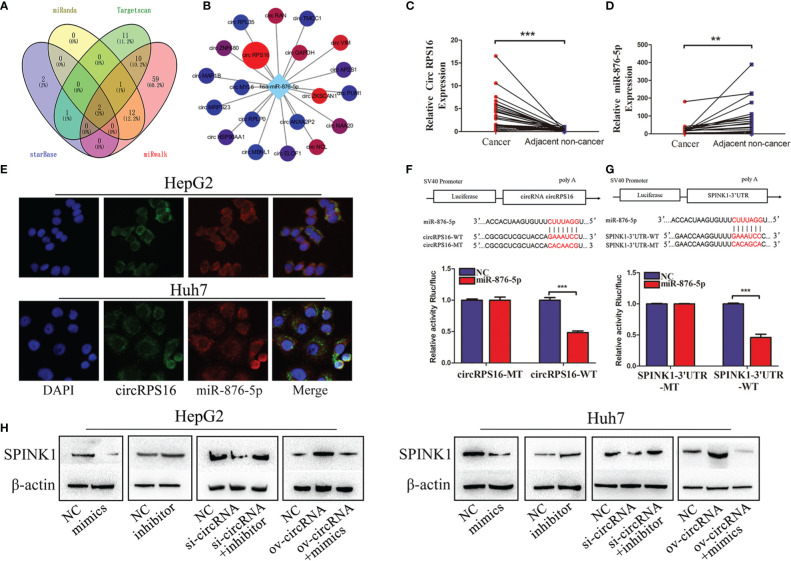
circRPS16 regulates SPINK1 by sponging miR-876-5p. **(A)** The potential miRNA targeting SPINK1 were predicted by miRanda, Targetscan, starbase, and miRwalk database; the interaction is shown in the Venn diagram. **(B)** The potential circRNAs targeting miR-876-5p were predicted by starbase. **(C)** The circRPS16 and **(D)** miR-876-5p expression levels were qualified by qRT-PCR. Data are presented as mean ± SD; statistical significance was assessed by paired *t*-test. **p < 0.01, ***p < 0.001. **(E)** The location of circRPS16 and miR-876-5p was determined by FISH. **(F)** Dual-luciferase reporter assays demonstrated that circRPS16 directly targets miR-876-5p and **(G)** miR-876-5p directly targets SPINK1. Independent experiments were repeated three times; data are presented as mean ± SD; statistical significance was assessed by Student’s *t*-test. ***p < 0.001. **(H)** Western blot analysis shows that circRPS16 regulated SPINK1 expression *via* miR-876-5p. ov, overexpressed plasmid vector; si, siRNA; NC, negative control.

Reports indicate that circular RNAs act as sponges for miRNAs modulating the activity of miRNA on their target genes (19, 20). Unambiguously, circRNAs are considered a large class of posttranscriptional regulators (21). Therefore, we investigated whether SPINK1 is regulated by circular RNA. As a result, miR-876-5p was a potential miRNA regulating SPINK1, which was predicted by four databases including miRanda, Targetscan, starbase, and miRwalk (**Figure 3A**). Subsequently, the starBase database was used to investigate the potential upstream circRNAs of miR-876-5p. The results revealed that circRPS16 (circBase ID is hsa_circ_0050997) was the most potential one to bind miR-876-5p, which was supported by 32 Argonaute (AGO) CLIP seq experiments (**Figure 3B**) (22). The qRT-PCR results further demonstrated that circRPS16 is upregulated in HCC tissues (**Figure 3C**), nevertheless, miR-876-5p was downregulated in HCC tissues (**Figure 3D**). The FISH analysis confirmed that circRPS16 and miR-876-5p are colocalized in the cytoplasm of HCC cells (**Figure 3E**). Dual-luciferase system analysis was conducted for further validation. In contrast with the mutant type of circRPS16 plasmid, cotransfection of miR-876-5p mimics and wild type of circRPS16 plasmid significantly reduced the luciferase activity (**Figure 3F**). Moreover, unlike the transfection of plasmid containing mutant type of SPINK1 3′UTR, cotransfection of miR-876-5p mimics with wild-type plasmid decreased the luciferase activity (**Figure 3G**). Western blot assay was performed to find out whether circRPS16 regulates SPINK1 expression *via* miR-876-5p. As a result, transfection of miR-876-5p mimics downregulated SPINK1 expression, while miR-876-5p inhibitor upregulated SPINK1 expression. For the rescue assay, circRPS16 siRNA downregulated SPINK1 expression; this was restored by miR-876-5p inhibitor. Overexpressed circRPS16 vector upregulated SPINK1 expression, which was impaired by miR-876-5p mimics (**Figure 3H**).

In summary, the above findings prove that circRPS16 regulates SPINK1 expression *via* miR-876-5p.

### miR-876-5p Regulates HCC Cell Proliferation, Cell Cycle, and Invasion

**Figure 4:**
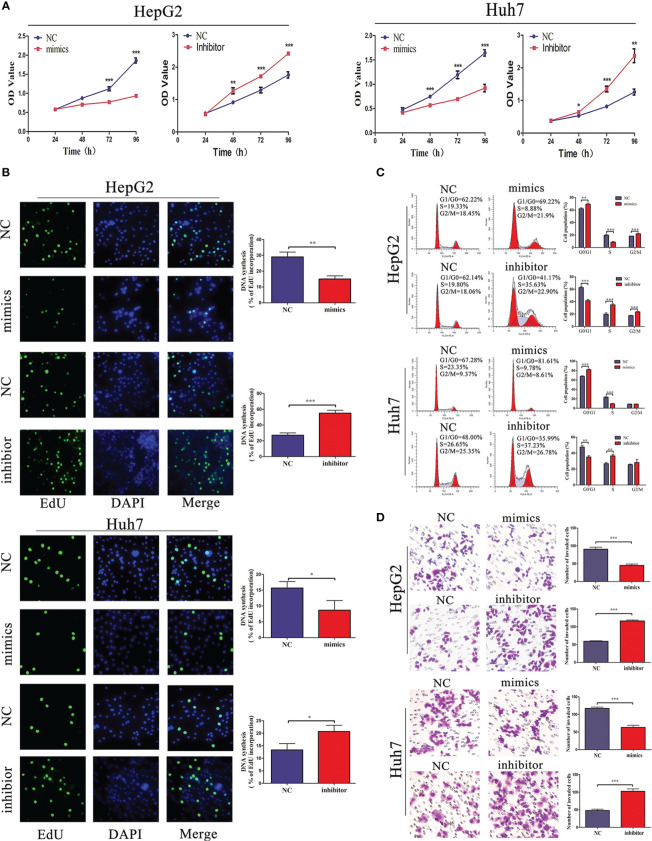
miR-876-5p regulates HCC cell proliferation, cell cycle, and invasion capacity. **(A)** CCK-8 and **(B)** EdU assays reveal that miR-876-5p mimics inhibited and inhibitor promoted the proliferation of HCC cells. **(C)** Flow cytometry reveals that miR-876-5p mimics induced cell cycle arrest, while, miR-876-5p inhibitor accelerated cell cycle. **(D)** Transwell assays reveal miR-876-5p mimics attenuated, while, inhibitor promoted the invasion of HCC cells. All the above experiments were repeated three times independently; data are presented as mean ± SD; statistical significance was assessed by Student’s *t*-test. *p < 0.05, **p < 0.01, ***p < 0.001.

Considering that miR-876-5p directly targeted SPINK1 and was downregulated in HCC tissues, its functional roles were further explored. miR-876-5p mimics or inhibitor was transfected to ectopically upregulate or downregulate miR-876-5p expression, respectively. Meanwhile, the relevant NC were also transfected. CCK-8 and EdU assays identified that, unlike the NC group, miR-876-5p mimics inhibited HCC cell proliferation. In contrast, miR-876-5p inhibitor promoted HCC cell proliferation (**Figures 4A, B** **)**. Additionally, flow cytometry analysis revealed that miR-876-5p mimics caused cell cycle arrest, while miR-876-5p inhibitor promoted cell cycle progression (**Figure 4C**). Transwell assays were further conducted to evaluate the effects of miR-876-5p on metastasis. In contrast with the NC group, miR-876-5p mimics inhibited the invasion capacity of HCC cells, whereas miR-876-5p inhibitor improved this capacity (**Figure 4D**). These results imply that miR-876-5p exerts inhibitory roles in HCC progression *in vitro*.

### circRPS16 Regulates HCC Cell Proliferation, Cell Cycle, and Invasion by Sponging miR-876-5p

**Figure 5:**
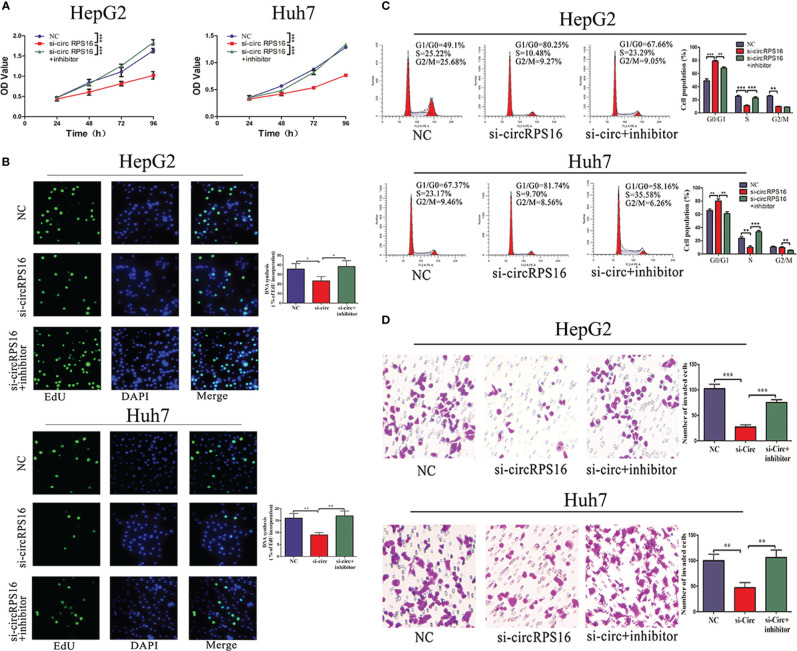
circRPS16 regulates HCC cell proliferation, cell cycle, and invasion capacity *via* miR-876-5p. **(A)** CCK-8 and **(B)** EdU assays show that circRPS16 knockdown inhibited the proliferation of HCC cells and miR-876-5p inhibitor restored the proliferation of circRPS16 knockdown HCC cells. **(C)** Flow cytometry reveals that circRPS16 knockdown induced cell cycle arrest and miR-876-5p inhibitor accelerated cell cycle of circRPS16 knockdown HCC cells. **(D)** Transwell assays show that circRPS16 knockdown inhibited invasion capacity of HCC cells and miR-876-5p inhibitor restored invasion capacity of circRPS16 knockdown HCC cells. All the above experiments were repeated three times independently; data are presented as mean ± SD; statistical significance was assessed by Student’s *t*-test. *p < 0.05, **p < 0.01, ***p < 0.001. NC, negative control; si, siRNA.

The independent roles of circRPS16 were confirmed through the transfection of circRPS16 siRNA. Then, to examine whether circRPS16 exerts regulatory roles by interacting with miR-876-5p, rescue assays were performed through cotransfection of circRPS16 siRNA and miR-876-5p inhibitor. CCK-8 and EdU assays showed that knockdown of circRPS16 inhibited HCC cell proliferation (**Figures 5A, B** **)**. Flow cytometry analyses demonstrated that circRPS16 siRNA caused cell cycle arrest, nonetheless (**Figure 5C**). Transwell assays identified that circRPS16 siRNA inhibited HCC cell invasion capacity (**Figure 5D**). Furthermore, rescue assays confirmed that reintroduction of miR-876-5p inhibitor rescued the inhibitory roles of circRPS16 siRNA on proliferation (**Figures 5A, B** **)**, cell cycle (**Figure 5C**), and invasion (**Figure 5D**). These results indicate that circRPS16 regulates HCC cell proliferation, cell cycle, and invasion by sponging miR-876-5p.

### Knockdown of circRPS16 Inhibits Tumor Growth by Suppressing SPINK1 *In Vivo*

**Figure 6:**
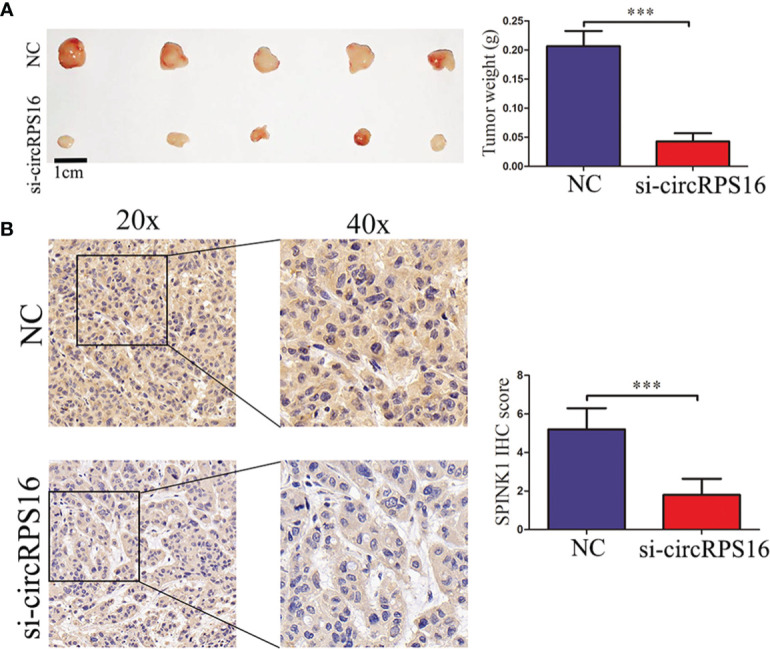
circRPS16 knockdown inhibits tumor growth *in vivo*. **(A)** After four weeks, the implanted mice were killed and the tumor weights were recorded. Data are presented as mean ± SD; statistical significance was assessed by Student’s *t*-test. ***p < 0.001. **(B)** The SPINK1 expression levels in each group were measured through IHC analysis. Data are presented as mean ± SD; statistical significance was assessed by Student’s *t*-test. ***p < 0.001. NC, negative control; si, lentiviral-circRPS16-RNAi.

To detect the roles of circRPS16 on HCC growth *in vivo*, HepG2 cells with stable knockdown of circRPS16 and the NC cells were implanted into the nude mouse model. After 20 days, the xenograft mice were euthanized and tumor weight was recorded. The results revealed that tumors derived from circRPS16 knockdown cells have a lower weight than those from NC cells (**Figure 6A**). To further assess whether circRPS16 regulates SPINK1 expression *in vivo*, SPINK1 expression levels of the different group was detected through IHC assay. The results demonstrated that SPINK1 expression was significantly downregulated in the circRPS16 knockdown group (**Figure 6B**). As such, *in vivo* assay confirms that circRPS16 knockdown inhibits SPINK1 and tumor growth.

## Discussion

HCC is a major international health problem; while acknowledging significant progress in its prevention, detection, diagnosis, and treatment, its incidence and mortality rates are increasing (1). Notably, molecular alterations regulate the transition of dysplastic cells to full-blown hepatocellular carcinoma (23). Therefore, targeting the molecular targets implicated in its pathogenesis is a potential treatment approach (24). Studies indicate that SPINK1 regulates HCC progression. For instance, SPINK1 differentiates a well-differentiated HCC from a high-grade dysplastic nodule (12). Moreover, SPINK1 is significantly upregulated in HCC tissues compared with corresponding nonmalignant tissues (25, 26). It also causes the epithelial-mesenchymal transition (EMT) *via* MEK/ERK pathway (27) and acts as a downstream effector of the CDH17/β-catenin axis in HCC (28). By analyzing the GEO database, we found that SPINK1 was upregulated in the HCC tissues; this was further verified using qRT-PCR in 25 paired HCC tumors and adjacent nontumors. TCGA data showed that high SPINK1 expression is closely associated with poor prognosis. In addition, IHC results from the Human Protein Atlas revealed that SPINK1 was upregulated in HCC tissues. Functional assays confirmed that SPINK1 acts as an HCC oncogene. These results indicate the vital roles of SPINK1 in HCC progression and are urgently required to elucidate its upregulatory mechanism.

circRNA is a novel class of noncoding RNA with a covalent closed-loop structure through back splicing (29). This special structure offers circRNA distinct characteristics such as inhibiting digestion and cleavage of exonucleases and long half-life compared with linear RNAs (30). Previous studies have reported the vital roles of circRNA in tumorigenesis and tumor progression including HCC (31, 32). To a certain degree, the locations of circRNAs dictate their functions. Nuclear retained circRNAs are implicated in transcription regulation (33, 34). Additionally, the circular RNAs retained in the cytoplasm act as miRNA sponges then regulate the expression of the miRNA target genes (4, 35). Furthermore, circRNAs form specific circRNPs by interacting with different proteins and translate into protein and form pseudogenes (19). Through bioinformatic analysis, we hypothesized that circRPS16 acts as a miR-876-5p sponge to regulate the SPINK1 expression. Previous studies have investigated the regulating roles of miR-876-5p in prostate cancer, breast cancer, HCC, etc. (36, 37). In HCC, miR-876-5p acts as the targets of LncRNA PITPNA-AS1, long noncoding RNA SNHG14, and LINC-ROR and then regulates proliferation and sorafenib sensitivity of HCC (38–40). miR-876-5p also suppresses HCC progression by targeting DNMT3A and inhibits EMT and metastasis of HCC by targeting BCL6 corepressor like 1 (BCORL1) (36, 41). These findings strongly indicate the essential roles of miR-876-5p in HCC. In our study, qRT-PCR results confirmed that circRPS16 was significantly upregulated; this was inversely associated with miR-876-5p expression in HCC tissues. FISH assays revealed that circRPS16 and miR-876-5p were colocalized in the cytoplasm. Dual-luciferase reporter assays proved that miR-876-5p directly combined circRPS16 and SPINK1. Subsequently, Western blot analysis showed that circRPS16 regulated SPINK1 expression *via* miR-876-5p. Therefore, the mechanism assays demonstrated that circRPS16 acts as the sponge of miR-876-5p, then regulate the miR-876-5p target gene SPINK1. Furthermore, functional assays confirmed the oncogenic roles of circRPS16 and the tumor-suppressor roles of miR-876-5p. Rescue assays verified that circRPS16 regulated HCC cell proliferation, cell cycle, and invasion *via* miR-876-5p. Eventually, our results show that circRPS16 inhibits tumor growth *via* SPINK1 *in vivo*.

In conclusion, our findings suggest that SPINK1 is upregulated in HCC tissues and acts as an oncogene in HCC progression. Furthermore, SPINK1 is regulated by circRPS16 *via* sponging miR-876-5p. To our knowledge, this is the first study reporting the important roles of circRPS16/miR-876-5p/SPINK1 axis in HCC progression. circRPS16 and SPINK1 are the potential diagnostic biomarkers and therapeutic targets for HCC. Thus, our findings provide additional insights into the molecular and regulatory mechanisms in the progression of HCC.

## Data Availability Statement

The datasets presented in this study can be found in online repositories. The names of the repository/repositories and accession number(s) can be found in the article/supplementary material.

## Ethics Statement

The studies involving human participants were reviewed and approved by the Research Ethics Committee, Guangdong Provincial People’s Hospital, Guangdong Academy of Medical Sciences. The patients/participants provided their written informed consent to participate in this study. The animal study was reviewed and approved by The Institutional Animal Care and Use Committee of the Guangdong Provincial People’s Hospital, Guangdong Academy of Medical Sciences.

## Author Contributions

ZJ contributed to study design, technical support, and revision of the manuscript. SL and YL contributed to study design, experiment implementation, data acquisition, statistical analysis, and the first draft of the manuscript. ZW, WX, and TP collected the specimen and performed experiments. ZZ, ZM, and LM performed bioinformatics analysis and statistical analysis. CJ provided conceptual advice. All authors contributed to the article and approved the submitted version.

## Funding

This work was supported by the Social Science and Technology Development Foundation of Dongguan (No. 202050715025194); National Natural Science Foundation of China (No. 81972792); National Science Foundation of Guangdong Province (No. 2020A1515010149).

## Conflict of Interest

The authors declare that the research was conducted in the absence of any commercial or financial relationships that could be construed as a potential conflict of interest.

## Publisher’s Note

All claims expressed in this article are solely those of the authors and do not necessarily represent those of their affiliated organizations, or those of the publisher, the editors and the reviewers. Any product that may be evaluated in this article, or claim that may be made by its manufacturer, is not guaranteed or endorsed by the publisher.

## Abbreviations

HCC, hepatocellular carcinoma; SPINK1, serine protease inhibitor Kazal type 1; FISH, fluorescent *in situ* hybridization; TATI, tumor-associated trypsin inhibitor; PSTI, pancreatic secretory trypsin inhibitor; GEO, Gene Expression Omnibus; ceRNA, competing endogenous RNAs; IHC, immunohistochemistry; ATCC, American Type Culture Collection; DMEM, Dulbecco’s modified Eagle’s medium; FBS, fetal bovine serum; qRT-PCR, quantitative reverse transcription PCR; CCK-8, Cell Counting Kit-8; PI, propidium iodide; NC, negative control; SD, standard deviation; TCGA, The Cancer Genome Atlas; AGO, Argonaute.
